# Temperate Bacteriophages from Chronic Pseudomonas aeruginosa Lung Infections Show Disease-Specific Changes in Host Range and Modulate Antimicrobial Susceptibility

**DOI:** 10.1128/mSystems.00191-18

**Published:** 2019-06-04

**Authors:** Mohammad A. Tariq, Francesca L. C. Everest, Lauren A. Cowley, Rosanna Wright, Giles S. Holt, Hazel Ingram, Liberty A. M. Duignan, Andrew Nelson, Clare V. Lanyon, Audrey Perry, John D. Perry, Stephen Bourke, Michael A. Brockhurst, Simon H. Bridge, Anthony De Soyza, Darren L. Smith

**Affiliations:** a Faculty of Health and Life Sciences, University of Northumbria, Newcastle upon Tyne, United Kingdom; b Department of Biology and Biochemistry, University of Bath, Bath, United Kingdom; c Department of Animal and Plant Sciences, University of Sheffield, Sheffield, United Kingdom; d Freeman Hospital, Newcastle upon Tyne, United Kingdom; e Royal Victoria Infirmary Hospital, Newcastle upon Tyne, United Kingdom; f Institute of Cellular Medicine, Newcastle University, Newcastle upon Tyne, United Kingdom; University of California, Irvine

**Keywords:** antimicrobial susceptibility, bacteriophages, bronchiectasis, cystic fibrosis, lysogenic, metagenomics, temperate

## Abstract

Temperate bacteriophages are a common feature of Pseudomonas aeruginosa genomes, but their role in chronic lung infections is poorly understood. This study was designed to identify the diverse communities of mobile P. aeruginosa phages by employing novel metagenomic methods, to determine cross infectivity, and to demonstrate the influence of phage infection on antimicrobial susceptibility. Mixed temperate phage populations were chemically mobilized from individual P. aeruginosa, isolated from patients with cystic fibrosis (CF) or bronchiectasis (BR). The infectivity phenotype of each temperate phage lysate was evaluated by performing a cross-infection screen against all bacterial isolates and tested for associations with clinical variables. We utilized metagenomic sequencing data generated for each phage lysate and developed a novel bioinformatic approach allowing resolution of individual temperate phage genomes. Finally, we used a subset of the temperate phages to infect P. aeruginosa PAO1 and tested the resulting lysogens for their susceptibility to antibiotics. Here, we resolved 105 temperate phage genomes from 94 lysates that phylogenetically clustered into 8 groups. We observed disease-specific phage infectivity profiles and found that phages induced from bacteria isolated from more advanced disease infected broader ranges of P. aeruginosa isolates. Importantly, when infecting PAO1 *in vitro* with 20 different phages, 8 influenced antimicrobial susceptibility. This study shows that P. aeruginosa isolated from CF and BR patients harbors diverse communities of inducible phages, with hierarchical infectivity profiles that relate to the progression of the disease. Temperate phage infection altered the antimicrobial susceptibility of PAO1 at subinhibitory concentrations of antibiotics, suggesting they may be precursory to antimicrobial resistance.

**IMPORTANCE** Pseudomonas aeruginosa is a key opportunistic respiratory pathogen in patients with cystic fibrosis and non-cystic fibrosis bronchiectasis. The genomes of these pathogens are enriched with mobile genetic elements including diverse temperate phages. While the temperate phages of the Liverpool epidemic strain have been shown to be active in the human lung and enhance fitness in a rat lung infection model, little is known about their mobilization more broadly across P. aeruginosa in chronic respiratory infection. Using a novel metagenomic approach, we identified eight groups of temperate phages that were mobilized from 94 clinical P. aeruginosa isolates. Temperate phages from P. aeruginosa isolated from more advanced disease showed high infectivity rates across a wide range of P. aeruginosa genotypes. Furthermore, we showed that multiple phages altered the susceptibility of PAO1 to antibiotics at subinhibitory concentrations.

## Abstract (precis)

Pseudomonas aeruginosa is a key opportunistic respiratory pathogen in patients with cystic fibrosis and non-cystic fibrosis bronchiectasis. The genomes of these pathogens are enriched with mobile genetic elements including diverse temperate phages. While the temperate phages of the Liverpool epidemic strain have been shown to be active in the human lung and enhance fitness in a rat lung infection model, little is known about their mobilization more broadly across P. aeruginosa in chronic respiratory infection. Using a novel metagenomic approach, we identified eight groups of temperate phages that were mobilized from 94 clinical P. aeruginosa isolates. Temperate phages from P. aeruginosa isolated from more advanced disease showed high infectivity rates across a wide range of P. aeruginosa genotypes. Furthermore, we showed that multiple phages altered the susceptibility of PAO1 to antibiotics at subinhibitory concentrations.

## INTRODUCTION

Cystic fibrosis (CF) and bronchiectasis (BR) are respiratory diseases that predispose the individual to chronic infection with opportunistic bacterial pathogens. Pseudomonas aeruginosa is a key pathogen in CF and BR that is associated with increased mortality (1, 2). P. aeruginosa can grow as a biofilm, which aids colonization and likely worsens outcomes in the chronically infected lung (3). The difficulty in effective delivery of antibiotics to the lung additionally compromises this treatment, and poor antimicrobial pharmacokinetics may contribute to the development of antimicrobial resistance (AMR) (1, 4). P. aeruginosa has a large and flexible genome including a complex accessory genome (5, 6). Comparative genomics shows that P. aeruginosa genomes contain a diversity of phage elements that represent a rich source of new genetic material, including antibiotic resistance genes that may be present and undergo frequent recombination (6–8). Therefore, temperate phages integrated into the bacterial host genome as a lysogen are likely to play an important roles in P. aeruginosa evolution as agents facilitating horizontal gene transfer (HGT) of cargo DNA and additional gene functions. However, changes in integrated phage genomes lead to deactivation of phage induction, and they are therefore no longer capable of lysis or onward HGT (9). Remaining phage gene regions may be a rich source of important gene function and a reservoir for mobilizing phage recombination. The fraction of integrated temperate phages in P. aeruginosa genomes that remain inducible is unknown, since this is rarely tested in comparative genomics studies, limiting our understanding of the potential for phage mobilization in chronic lung infections.

Previous work on the Liverpool epidemic strain (LES) of P. aeruginosa, which was isolated from a CF patient, has shown that this strain harbors multiple inducible temperate phages in its genome (10). These phages remain active and capable of lysis even after many years of chronic CF lung infection, suggesting that they may play an important role in the CF lung (11). Experimental infections in both insect and mammalian hosts demonstrate that the LES temperate phages increase the fitness of P. aeruginosa lysogens *in vivo*, through phage-mediated killing of competing P. aeruginosa strains (12, 13). Furthermore, P. aeruginosa is known to adapt to the lung environment during chronic infection leading to loss of motility (14), mucoid phenotype (15), and biofilm formation (16, 17). The LES temperate phages have been shown to contribute to this P. aeruginosa evolutionary adaptation in a sputum-like medium by causing phage insertion-mediated beneficial mutations, particularly in motility and quorum-sensing-associated genes (18). Given these potentially important contributions to P. aeruginosa, we need to understand the potential for phage mobilization across a wider range of clinical P. aeruginosa strains.

Separating individual phage genomes from meta-data is challenging, as related phages share genomic similarity, which increases the probability of assembling chimeric phages. This currently means that it is difficult to characterize phage species at the genome resolution in a mixed population, which requires isolation and propagation of individual phages before genomic comparison and characterization can occur. This can be complicated further by the lack of a receptive bacterial host. We therefore used a novel metagenomic approach and k-mer abundance strategy to resolve individual temperate phages that are mobilizing in complexes from P. aeruginosa genomes. This approach uses the differences in numbers of released phages from the cell during chemical induction and targets these differences in copy number of phage genomes. Here, we characterized the diversity of temperate phages induced from a large panel of P. aeruginosa isolates isolated from patients with either CF or BR. The mobilizable phages were compared at the genomic level, and infectivity profiles were used to determine the similarity and differences between CF and BR. Furthermore, we evaluated the effects of temperate phage infection on the antimicrobial susceptibility of P. aeruginosa *in vitro*.

## RESULTS

### Phage infectivity profiles.

**FIG 1:**
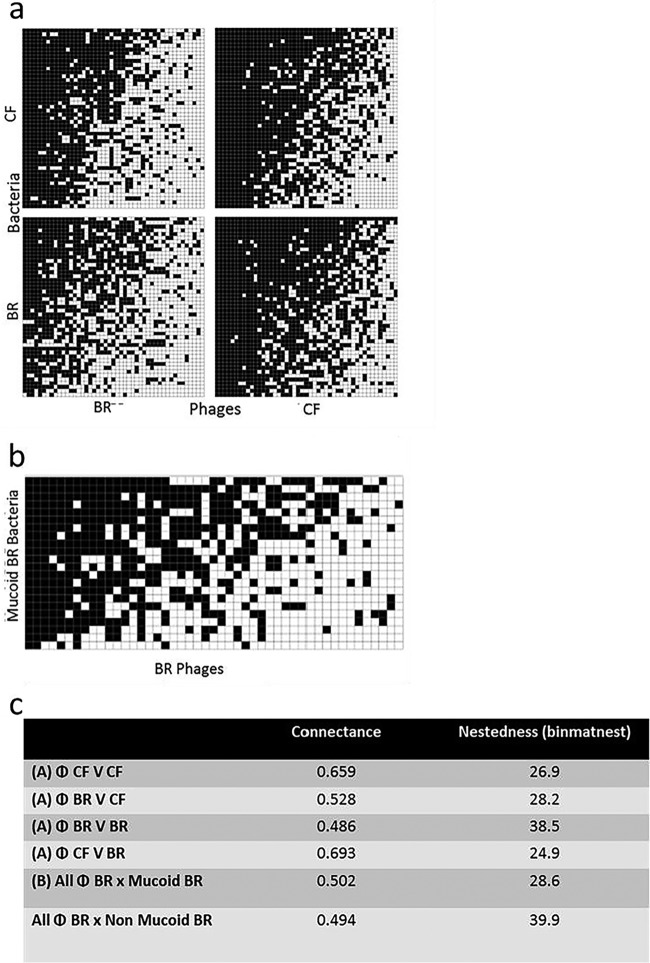
Shows nestedness and connectance plots demonstrating that temperate phages from P. aeruginosa isolated from patients with advanced respiratory infection can infect a wider range of P. aeruginosa genotypes. The nestedness and connectance plots map the cross-infection data from mixed temperate phage communities induced from clinical P. aeruginosa isolates. (A) The binary nested network for the complete 94 phage lysates against 94 P. aeruginosa isolates, ordered by nestedness within equally sized quadrants (47 CF phages versus 47 CF P. aeruginosa isolates, 47 BR phages versus 47 CF P. aeruginosa isolates, 47 CF phages versus 47 BR P. aeruginosa isolates, and 47 BR phages versus 47 BR P. aeruginosa isolates). Each black square represents an individual interaction (infection event) between one phage strain (*x* axis) and one bacterial strain (*y* axis). White squares represent lack of infection. Note the CF-derived phages are capable of infecting BR P. aeruginosa isolates more frequently than BR phages can infect CF P. aeruginosa, illustrated through higher values for connectance (0.693). (B) The binary nested network representation of infection of the mucoid BR P. aeruginosa isolates (*n* = 22) with the entire cohort of mixed temperate phage communities induced from BR P. aeruginosa isolates (*n* = 47). (C) Nestedness and connectance values for the results shown in panels A and B.

Lambdoid-like phages were chemically induced from each P. aeruginosa isolate using the fluoroquinolone antibiotic norfloxacin. Importantly, all the clinical P. aeruginosa isolates in this panel contained inducible prophages, which is a higher proportion than previously reported in other Gram-negative bacteria such as Escherichia coli (19). Binary networks of phage lysate infectivity against all P. aeruginosa isolates revealed that CF phages are more broadly infectious than BR phages (i.e., higher connectance) (Fig. 1A and C). However, interestingly, binary networks of BR phage lysates (*n* = 47) had a more nested structure with higher connectance against mucoid BR isolates (*n* = 22) (Fig. 1B and C), suggesting that these phages were better able to infect mucoid phenotypes than nonmucoid P. aeruginosa strains. Semisupervised data reduction modeling (PLS-DA) suggested that the phage lysate infectivity range against P. aeruginosa was greater for phages induced from P. aeruginosa isolated from more advanced stages of disease in both CF and BR (see Fig. S1 in the supplemental material). This allowed stratification of the phage lysates into “early” and “late” disease stages (CF patients were divided into those under the age 16 years or over age 16; BR patients were divided into those less than 10 years since diagnosis or more than 10 years since diagnosis) for subsequent analyses.

10.1128/mSystems.00191-18.1 FIG S1 PLS-DA plots showing the variation in phage infectivity, semisupervised with the associated discriminating clinical information. (A) Phage infectivity profiles being studied for both subdivisions of the CF isolates (*R*^2^*Y* is 82.5%); adult CF, blue; pediatric CF, red. (B) Phage infectivity profiles when the BR isolates are stratified according to length of time since diagnosis (*R*^2^*Y* is 40%). <5, yellow; >5 and <10 years, light blue; >10 years, dark blue. Download FIG S1, TIF file, 1.3 MB. Copyright © 2019 Tariq et al. 2019 Tariq et al. This content is distributed under the terms of the Creative Commons Attribution 4.0 International license.

### Isolating single phage genomes from mixed phage populations induced from a clonal bacterial culture using k-mer count and abundance.

We used the biology of temperate phage life cycles and the presumed differences in burst size and diversity to enable 105 complete individual phage genomes to be resolved from the DNA sequencing data. With various numbers of phages in each mixed lysate, a method was implemented to transform the data to distinguish between copy numbers of different viral genomes to resolve individual phages. The Khmer analysis toolkit (20) uses k-mer count and abundance to separate data and is normally used to determine the error k-mer that hinders genome assembly. We modified the python script (calc_median_distribution.py) in Khmer to allow extraction of individual phage sequences that could be assembled into single nonchimeric genomes (see the complete method in Text S1 in the supplemental material) using k-mer distribution. The workflow used to assemble each phage is summarized in Fig. S2.

10.1128/mSystems.00191-18.2 FIG S2 Summarizes the workflow approach used to assemble temperate phages from a mixed lysate sample. To assemble individual phage genomes from mixed viral communities, higher *N*_50_ scores for assembly were achieved using workflow 2; therefore, this approach was employed to generate the data in this study. Download FIG S2, TIF file, 1.7 MB. Copyright © 2019 Tariq et al. 2019 Tariq et al. This content is distributed under the terms of the Creative Commons Attribution 4.0 International license.

10.1128/mSystems.00191-18.8 TEXT S1 Supplemental methods. Download Text S1, DOCX file, 0.02 MB. Copyright © 2019 Tariq et al. 2019 Tariq et al. This content is distributed under the terms of the Creative Commons Attribution 4.0 International license.

### Analysis of P. aeruginosa phages in CF and BR.

**FIG 2:**
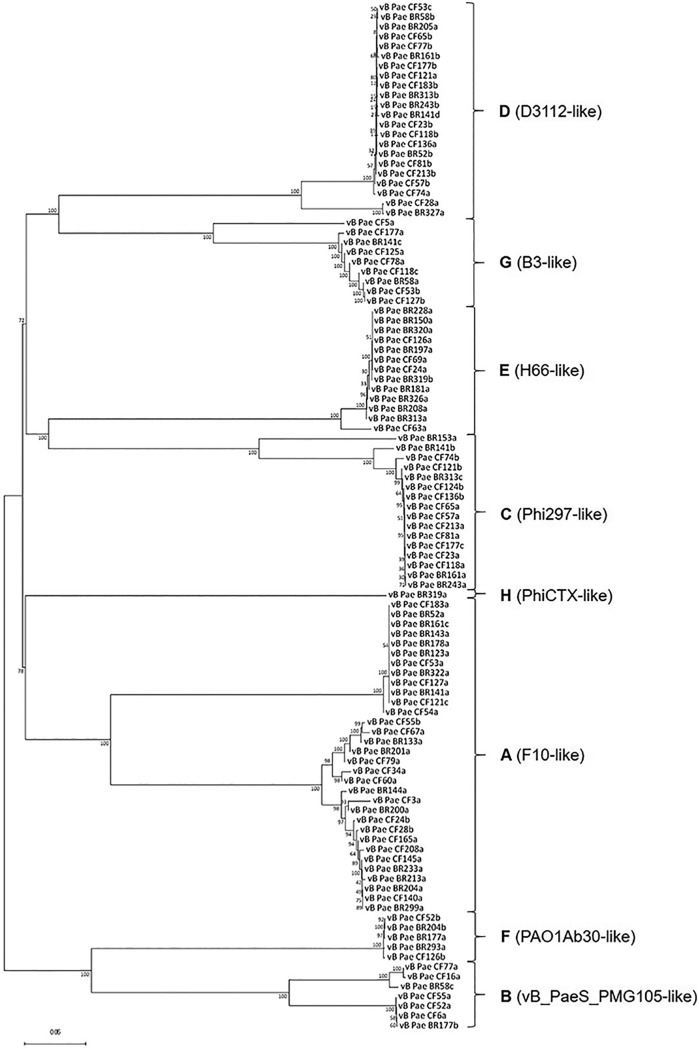
Phylogenetic tree of P. aeruginosa phages. The genome assemblies of the 105 P. aeruginosa phages were aligned using MAFFT. The accuracy method L-INS-i algorithm was used. The phages, grouped by sequence similarity, offer eight clades (A to H). The evolutionary history was inferred using the neighbor-joining method, with the sum of branch length of the optimal tree of 3.04826762. The percentages of replicate trees in which the associated taxa clustered together in the bootstrap test (1,000 replicates) are shown next to the branches; the tree was rooted at the midpoint. The evolutionary distances were computed using the *p*-distance method and are in the units of the number of base differences per site. All ambiguous positions were removed for each sequence pair. There were a total of 129,881 positions in the data set. Evolutionary analyses were conducted in MEGA7.

Each of the 105 phages was assigned to one of 8 groups (A to H) using a genome comparison dot plot method, Gepard (21) (see Fig. S3). The phage genomes were ordered based on genome similarity and disease etiology. Figure 2 shows whole-genome phylogenetic comparisons aligned with MAFFT, using the L-INS-i algorithm for higher accuracy, which also shows phylogenetic clustering into 8 groups. Basic local alignment (BLAST) was used to determine the closest relative within each phylogenetic clade which was labeled accordingly.

10.1128/mSystems.00191-18.3 FIG S3 Nucleotide dot plot of phage genomes. Nucleotide matrices comparing the sequence of 105 P. aeruginosa phages. The *x* and *y* axes consist of 105 phages that were concatenated together. This map was generated with Gepard v1.40 using a window size of 25 and word length of 10. The darker the area, the similar the sequence; a diagonal line represents a region of high genome similarity. Download FIG S3, TIF file, 2.8 MB. Copyright © 2019 Tariq et al. 2019 Tariq et al. This content is distributed under the terms of the Creative Commons Attribution 4.0 International license.

A key aim of this study was to report individual phage genomes; therefore, we only included single contiguous sequence assemblies in downstream analyses (see Table S1). The stringency of our selection criteria removed any phages with fragmented assemblies or low sequence coverage to allow single phage genome comparisons (for partial phage genomes identified within the data, see Table S1). Taxonomic assignment of assembled genomes indicated that *Caudovirales* phages were enriched in P. aeruginosa isolates in CF and BR (Fig. 2). Groups A to D, F, and G were related to the *Siphoviridae* family (*n* = 91), group E was related to the *Podoviridae* family (*n* = 13), and group H to the *Myoviridae* family (*n* = 1). The distributions of phages between the groups (A to H) and disease etiologies are shown Table S2.

10.1128/mSystems.00191-18.5 TABLE S1 Summarizing the number of k-mer peaks, assemblies, and phages resolved. Shown are percentages infectivity of each phage complex across the panel of 94 Pseudomonas aeruginosa isolates and phage genome accession numbers. *, phages that were resolved using >10 sequence coverage and nonchimeric; †, partial phages and low sequence coverage assemblies. Download Table S1, DOCX file, 0.02 MB. Copyright © 2019 Tariq et al. 2019 Tariq et al. This content is distributed under the terms of the Creative Commons Attribution 4.0 International license.

10.1128/mSystems.00191-18.6 TABLE S2 Phage genotype distribution between CF and BR. Data within brackets show incomplete or partially resolved phage genomes. Download Table S2, DOCX file, 0.01 MB. Copyright © 2019 Tariq et al. 2019 Tariq et al. This content is distributed under the terms of the Creative Commons Attribution 4.0 International license.

Tables S1 and S2 show both complete and partial genomes determined from the data. We identified phage group A (F10 like) as the most frequently found group (*n* = 32) in both disease backgrounds. Group E phages (H66/F116 like) were commonly identified in BR. In contrast, group D (D3112-like), C (Phi 297-like), and group G (B3-like) phages were more frequently found in CF. The Phi297-like phages show genome homology to D3 and F116, and this is shown in the Gepard plot (Fig. S3) (22). Interestingly group D phages (D3112 like) (Mu-like transposable phages) (23) and group C (Phi297 like) were found in the same P. aeruginosa metaviromes, suggesting a possible co-lineage. We also identified a cytotoxin-converting PhiCTX-like phage (group H), which carries the CTX gene; this phage was found in a single BR P. aeruginosa isolate (24).

### Antimicrobial susceptibility of twenty PAO1 lysogens.

**FIG 3:**
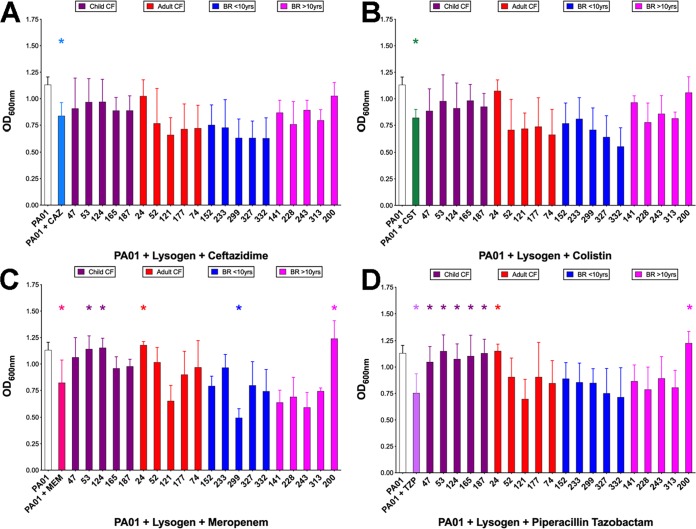
Antimicrobial susceptibility of lysogens to clinically relevant antibiotics. The data shown are after 9 h of incubation: ceftazidime, 0.08 μg/ml (A); colistin, 1.6 μg/ml (B); meropenem, 0.08 μg/ml (C); and piperacillin, 0.8 μg/ml (D). *, *P* ≤ 0.05 by one-way ANOVA with Dunnet’s *post hoc* test for parametric variables or a one-way ANOVA Kruskal-Wallis test with Dunn’s multiple-comparison test. The lysogens were grouped (and color coded) based on disease etiologies.

From each clinical group (CF, <16 years of age, >16 years; BR, <10 and >10 years since diagnosis), 5 phage lysates were randomly selected to create P. aeruginosa PAO1 lysogens. Of the 20 lysogens, 8 showed altered antimicrobial susceptibility to clinically relevant antimicrobials compared to that of wild-type PAO1 when testing a concentration subinhibitory to the MIC for PAO1. Figure 3 shows the altered phage-mediated growth of PAO1 in the presence/absence of ceftazidime (Fig. 3A), colistin (Fig. 3B), meropenem (Fig. 3C), and piperacillin (Fig. 3D) (25). Seven lysogens significantly decreased the inhibition of growth to meropenem and piperacillin; one significantly increased inhibition of growth to meropenem. Antibiotic sensitivity was also compared to disease etiology, with only childhood CF phage PAO1 lysogens showing significant reduction in sensitivity to meropenem and piperacillin (see Fig. S4). The genomes of the 20 lysogens were sequenced and compared to that of wild-type PAO1 using Mauve (26) to determine phage integration sites (see Table S3). Importantly, we found that none of the phages integrated within putative genes that could be associated with this phenotype. The specific phage integrated into each site in the PAO1 chromosome is also detailed in Table S3.

10.1128/mSystems.00191-18.4 FIG S4 Comparisons of antimicrobial susceptibility of PAO1 and disease-specific lysogens to clinically relevant antibiotics. The OD_600_s of PAO1 growth after 9 h incubation (*n* = 9 replicates): ceftazidime, 0.08 μg/ml (A); colistin, 1.6 μg/ml (B); meropenem, 0.08 μg/ml (C); and piperacillin, 0.8 μg/ml (D). Boxplots (with Tukey ranges shown by the whiskers) compare the groups. Data points (closed circles) show the outliers. *, *P* ≤ 0.05 by one-way ANOVA with Dunnet’s *post hoc* test for parametric variables or a one-way ANOVA Kruskal-Wallis test with Dunn’s multiple-comparison test. The lysogens were grouped based on disease etiology (child CF, adult CF, BR < 10 years, and BR > 10 years). Download FIG S4, TIF file, 1.1 MB. Copyright © 2019 Tariq et al. 2019 Tariq et al. This content is distributed under the terms of the Creative Commons Attribution 4.0 International license.

10.1128/mSystems.00191-18.7 TABLE S3 P. aeruginosa integration site of the 20 prophages used for the antimicrobial resistance profiling of lysogens. The insertion site is usually between a hypothetical and tyrosine tRNA ligase gene. All phages integrated outside a putative coding region. Download Table S3, DOCX file, 0.01 MB. Copyright © 2019 Tariq et al. 2019 Tariq et al. This content is distributed under the terms of the Creative Commons Attribution 4.0 International license.

### Phage genome search for AMR gene prediction.

The 105 phage genomes were searched using 2 databases (CARD [27] and ARDB [28]) for known and previously reported AMR genes; none were identified in the phage genomes.

## DISCUSSION

This is the largest pan-genome study of P. aeruginosa temperate phages, which mapped the interactions between phage and bacteria and their broad infectivity across chronic respiratory disease-related P. aeruginosa isolates. We investigated a broad range of P. aeruginosa isolates from patients at different stages of disease, and to our knowledge, this is the largest reported cross-infection study of lysogenic phages undertaken for clinically derived P. aeruginosa isolates, with over 8,800 infections. We first showed that P. aeruginosa isolates from these diverse clinical sources have high levels of inducible phages compared to those of other Gram-negative bacteria (19). This illustrates a significant reservoir of mobile DNA that could play a major role in bacterial adaptation and evolution in the lung. This finding combined with a recent study by Nguyen and colleagues (29) demonstrating that phages are able to interact and translocate across epithelial surfaces adds a further layer of largely unexplored complexity to chronic respiratory infections.

We found a positive correlation between phage infectivity and disease progression in both CF and BR, which supports a study by James and colleagues that followed LES phage populations in LES-infected CF patients over a period of 28 months (11). Our approach here is more representative of phage induction in the lung, as it utilizes the infectivity of all the temperate phages, mobilized from each P. aeruginosa genome under selective pressure. This provides a panoramic snapshot of phage mobilization *in vivo* that may illustrate the broad reach of HGT in chronic respiratory disease.

One of our key findings is the hierarchical infectivity of CF- and BR-derived phage lysates by bipartite ecological network modeling. We saw that chronicity of infection was associated with greater levels of infectivity. These infectivity profiles supervised by age and time in CF and BR, respectively, show significant differences that allowed us to stratify this cross-sectional panel into 2 groups per disease. Previous studies have demonstrated that P. aeruginosa isolated from CF patients can cross-infect patients already infected with other P. aeruginosa (30–32), in particular, the Liverpool epidemic strain (LES). We have previously shown using Kyoto Encyclopedia of Genes and Genomes (KEGG) pathway analysis of these phage metagenomes that the size of the phage accessory genome increased with the progression of disease. Furthermore, we identified gene markers that were associated with survival in CF or BR patients with chronic P. aeruginosa infections (33). Through classifying these phage-host interactions, we showed the breadth of HGT that would occur on phage induction where we have previously described the function that can be disseminated directly to the bacterial host from the phage metagenomes (33). Similar studies have found that temperate phages mediate pathogen fitness in the lungs of CF patients and demonstrate their clinical importance in the pathophysiology of the disease (10, 11, 18). The broad infectivity of phage lysates derived from adult CF patients when infecting P. aeruginosa from other clinical sources suggests there are additional risks beyond our current understanding of “bacterial cross-infection,” where phage-mediated HGT adds another level of complexity.

Importantly, we developed a novel method for resolving individual phage genomes from metaviromes by modifying a program (Khmer [20]) used to remove the error k-mer from genome sequencing data. This method has allowed resolution of a variety of phage genotypes present in chronic respiratory disease-related P. aeruginosa. Given the importance of transposable phages in driving adaptive evolution of bacterial pathogens within hosts, the findings of this study highlight a number of temperate phages to focus on in prospective studies. For example, the group A or F10-like phages were the most commonly found and more diverse group (*n* = 32) across this panel of P. aeruginosa; however, despite their prevalence, there is currently limited research on the biology of these temperate phage (34). Other noteworthy examples include F116-like phages (group E); these have been shown to easily exchange bacterial chromosome genes (35), suggesting it may have a role in bacterial adaptation in the chronically infected lung. Phi297 has been described as producing halo-morphologies and has a narrow host range (22); in this study, the Phi297-like phages were predominantly found in CF isolates and more likely to be associated with a broader infectivity profile.

The phage genomes from group D (D3112 like) and group C (Phi297 like) in our cohort were intriguingly detected together, a marker that in tandem, they are potentially ecologically important, displaying a symbiotic relationship. We tested this further, and even when full contiguous sequences of one phages were present, the secondary phage was always found within the other k-mer peak assemblies.

Kim et al. showed that if P. aeruginosa develops resistance to PA1Ø phage, which shares genome homology to D3112 (a pilus-infecting phage), then biofilm formation was reduced, which leads to increased antibiotic susceptibility (36). This provides evidence that suggests that cocarriage of phages is required and may constitute an evolutionary advantage, something that has not been previously shown in P. aeruginosa. It is noteworthy that even though each clade relates to a previously described phage infecting P. aeruginosa, the genomic diversity within each phage group is extremely high and requires further study.

Current research focuses on identifying genes in bacterial backgrounds and studying the incidence of AMR, which is a key problem in P. aeruginosa infection in CF and BR. In this study, we demonstrate that phages modulate the growth of the lab strain PAO1 in the presence of antimicrobials and in the absence of known resistance genes. We show that 7 of the 20 phages decreased the susceptibility of P. aeruginosa lab strain PAO1 against one or more of the clinically relevant antipseudomonals and that 1 increased the susceptibility of P. aeruginosa at concentrations below the clinical MIC for PAO1; yet, the concentration of the antibiotic inhibited the growth of PAO1. Previous work by Fothergill et al. illustrated differential induction rates and, in some cases, reduction of phage release when LES P. aeruginosa isolates were challenged with commonly prescribed antibiotics (37). Microbiome studies of the gut have shown that antibiotic treatment enriched the phage metagenome and increased transfer of AMR genes under conditions of physiological stress (38, 39). Importantly we found no gene(s) associated with these small but significant changes in bacterial susceptibility to antibiotics using curated CARD and ARDB database searching. We therefore hypothesize that this association is mediated by phages and that they subvert normal cellular and metabolic pathways of P. aeruginosa. This was previously described for phages transferring genes horizontally in Escherichia coli (40). Here, we showed that phages 24 (CF) and 200 (BR) when integrated into PAO1 as lysogens have the greatest impact reducing the susceptibility to the antibiotics tested. We propose that a decrease in susceptibility may increase tolerance, which in turn may progress to AMR (41). Phage 24 is group A (F10 like) and the most heterogeneous compared to the other F10-like phages isolated here. Similarly, phage 200 is group D (D3112) and integrates at a different point in the genome than other group D phages. Observationally, phages associated with earlier stages of disease in CF provide P. aeruginosa with an increased resistance to antibiotic therapy. Does this mean that these phages are supportive in early colonization of the CF lung?

To date, there are no treatment algorithms sufficient to meet the demands of these new clinical challenges. The ability of phages to subvert the physiology of P. aeruginosa is worrying, especially given the lack of an associated gene target available for molecular testing. This study highlights an unmet need for further temperate phage P. aeruginosa studies in patients with CF and BR to understand how phage communities subvert their bacterial host and impact both microbial fitness and the composition of the lung microbiota.

## MATERIALS AND METHODS

### Patient selection.

Patients (*n* = 94; 47 cystic fibrosis and 47 non-CF bronchiectasis) were randomly chosen that were persistently infected with P. aeruginosa and attending specialist clinics at the Newcastle upon Tyne Hospitals NHS Trust. Ethical approval was obtained from County Durham and Tees Valley Research ethics committee (REC reference, 12/NE/0248).

### Norfloxacin induction of temperate phages.

Temperate phages were chemically induced from bacterial isolates using norfloxacin according to the method previously described (33, 42, 43).

### Bacteriophage infectivity assay.

Mid-exponential growth phase P. aeruginosa (100 μl) was added to 5 ml 0.4% (wt/vol) LB agar (high clarity agar [Lab M Limited, Heywood, UK] and 1% [wt/vol] 1 M CaCl_2_·2H_2_O [Sigma-Aldrich, Gillingham, UK]) and overlaid onto LB agar 1.5% (wt/vol). Each phage lysate was serially diluted and spotted onto a lawn of each P. aeruginosa isolate. The plates were incubated at 37°C for 18 h, and the various plaque morphologies were assessed. If a zone of clearing was seen, the lysates were diluted to define individual plaques to negate clearing due to pyocins.

### Modelling of cross-infection data.

Bipartite infection networks of bacteria and phage interactions both within and between diseases were assessed (CF phage versus CF P. aeruginosa, CF phage versus BR P. aeruginosa, BR phage versus CF P. aeruginosa, and BR phage versus BR P. aeruginosa). Nestedness was measured using the binary matrix nestedness temperature calculator (binmatnest) (44) within the “nestedness” metric from the R package bipartite v 2.04 (45, 46). All the binmatnest values given were statistically significant compared to the associated null model analysis. Network visualizations were generated using R package bipartite v 2.04. Nestedness is a measure of network order, where a highly nested pattern describes a hierarchy from generalists (broad host range) to specialists (narrow host range) for both groups. The binmatnest value is the deviation of the network pattern from an optimal nested pattern of the same dimensions; values are on a scale from 0 (highly nested) to 100 (not nested at all). To give these models numerical significance, the nestedness of the interactions was determined.

### Construction of a phylogenetic tree.

The 105 genome sequences were concatenated into a single fasta format file and aligned using MAFFT v7 (47). The L-INS-i algorithm was used to improve accuracy. The alignment file was used to create a *p*-distance analysis in MEGA7 (48) following construction of a neighbor-joining tree on *p*-distance using 1,000 bootstrap analyses.

### Generation of twenty P. aeruginosa PAO1 lysogens.

Twenty lysogens were generated using 5 randomly chosen phage lysates from each clinical grouping (see patient disease stratification in supplemental material) and used to infect PAO1, using an approximate multiplicity of infection of 0.1 (bacteria to phage, 10:1). Infections were incubated at 37°C for 30 min. Ten microliters of the incubated culture was inoculated on LB plates and incubated for 18 h at 37°C. Lysogeny was determined through phage induction from the resulting bacterial colonies and genome sequencing (Illumina, as per phage genome sequencing) and assembly through *de novo* reference assemblies using Ragout. The phage integration sites were tracked using Mauve (26).

### Antimicrobial susceptibility profiling of phage-infected PAO1.

In conformance with BSAC testing for *Pseudomonas* (adapted from reference 49), a 2% inoculum of P. aeruginosa culture with an optical density at 600 nm (OD_600_) of 0.03 in a 1:1 ratio with the antibiotics (total volume, 150 μl) was used in the assay. The antipseudomonal antimicrobials used were ceftazidime (Sigma-Aldrich), colistin (Forest Laboratories, London, UK), meropenem (Fresenius Kabi, Runcorn, UK), and piperacillin (Bowmed Ibisqus, Wrexham, UK). All tests were completed at subinhibitory concentrations of these antibiotics to compare between the PAO1 and each lysogen. Microtiter plates were incubated at 37°C, 50 rpm to limit biofilm formation and covered with a breathable seal (AeraSeal Sterile; Alpha Laboratories, Eastleigh, UK). Absorbance readings (OD_600_) were taken for the individual wells after 9 h of incubation.

### Statistical analyses.

All statistical tests were performed with the use of GraphPad Prism 8 (GraphPad, La Jolla, CA, USA). The distribution of data was determined from a *P* value of >0.05 using the Anderson-Darling test. Variations in antibiotic sensitivity between lysogen, PAO1, and antibiotic were analyzed using a one-way analysis of variance (ANOVA) with Dunnett’s *post hoc* test for parametric variables or the one-way ANOVA Kruskal-Wallis test with Dunn’s multiple-comparisons test for nonparametric variables. For all analyses, *P* values of <0.05 were considered significant.

### Phage DNA isolation.

Following confirmation of temperate phage presence from the infectivity assay, the lysate was treated with Turbo DNase (2 U) and RNase (100 U) (Life Technologies Limited) to remove bacterial chromosomal DNA and incubated at 37°C for 30 min. The enzymes were heat inactivated at 65°C at a final concentration of 15 mM EDTA for 10 min. Norgen Phage DNA isolation kits (Geneflow Limited, Lichfield, UK) were used to purify the viral DNA.

### Genome sequencing.

The Illumina Nextera XT (Illumina, Saffron Walden, UK) library preparation kit was used to prepare 94 individual sequencing libraries relating to each induction and phage DNA extraction; sequencing was completed using the Illumina MiSeq version 2, 2 × 250-cycle chemistry. Paired-end sequencing reads were provided as FASTQ files (NU-OMICS, Northumbria University at Newcastle, UK). The raw reads had their adapters removed using Trimmomatic (50) and were quality trimmed using Sickle at –q 30 and –l 15 (51).

### k-mer-based sequence separation.

The Velvet *de novo* genome assembler package v1.2.10 shuffleSequences_fastq.pl script was used to randomly shuffle the fastq sequences to limit bias. The shuffled sequence output file was directly pipelined into the Khmer toolkit v1.1, which was used for its designed ability to remove very low-level bacterial contamination and erroneous k-mers from the viral sequence data. The Khmer count table and k-mer abundance histogram clusters low abundance and poor sequence data that would be linked to any residual bacterial chromosomal DNA (20). The Khmer generated k-mer abundance data were graphed in Excel, and the error k-mer peak was manually selected and removed. The calc-median-distribution.py script part of Khmer (20) was modified and renamed k-mer_extraction.py (https://github.com/rmadnantariq/k-mer_extraction). The script was altered to select out the k-mers associated with an abundance peak distribution described in detail in the methods in the supplemental material.

### Phage genome assembly.

SPAdes v3.5.0 was used to assemble phage genomes using the careful parameter, utilizing kmer lengths 21, 33, 55, 77, 99, and 127 (52). The assembled contigs that were identified as partial were extended using Paired-Read Iterative Contig Extension assembler (PRICE v1.2) (53). PriceTI was invoked with a minimum overlap of 30 nucleotides using 90% high-quality reads with 90% identity for 51 cycles. The apc script was used to identify circular genomes and remove overlaps when positive (54), and putatively complete phage genomes were annotated using RASTtk (55–57).

### Nucleotide dot plot analysis using Gepard

The 105 phage genomes were concatenated together based on genome similarity and ordered based on disease etiologies. The dot plot was generated with Gepard (21) using a word size of 10 and sliding window of 25.

### Lysogen assembly.

CLC genomic workbench v11 was used to generate the assembly of the naive PAO1 lab strain. This assembly was used as a reference in Ragout post *de novo* assembly of the lysogens using SPAdes (v3.5.0). The assemblies were annotated using Prokka v1.22. The naive PAO1 and reference-assembled PAO1 lysogens were compared using Mauve to track the phage and their insertion sites.

### Data availability.

All data generated or analyzed during this study are included in the published article (and its supplemental material files). The raw fastq files were deposited in GenBank with the BioProject accession PRJNA503342. Resolved 105 phage genome accession numbers can be found in Table S1.
