# Supplementary figures and images for: Temperate Bacteriophages from Chronic Pseudomonas aeruginosa Lung Infections Show Disease-Specific Changes in Host Range and Modulate Antimicrobial Susceptibility
Source: mSystems. 2019 Jun 4;4(4):e00191-18. doi: 10.1128/mSystems.00191-18 (PMC6550368; doi:10.1128/mSystems.00191-18)

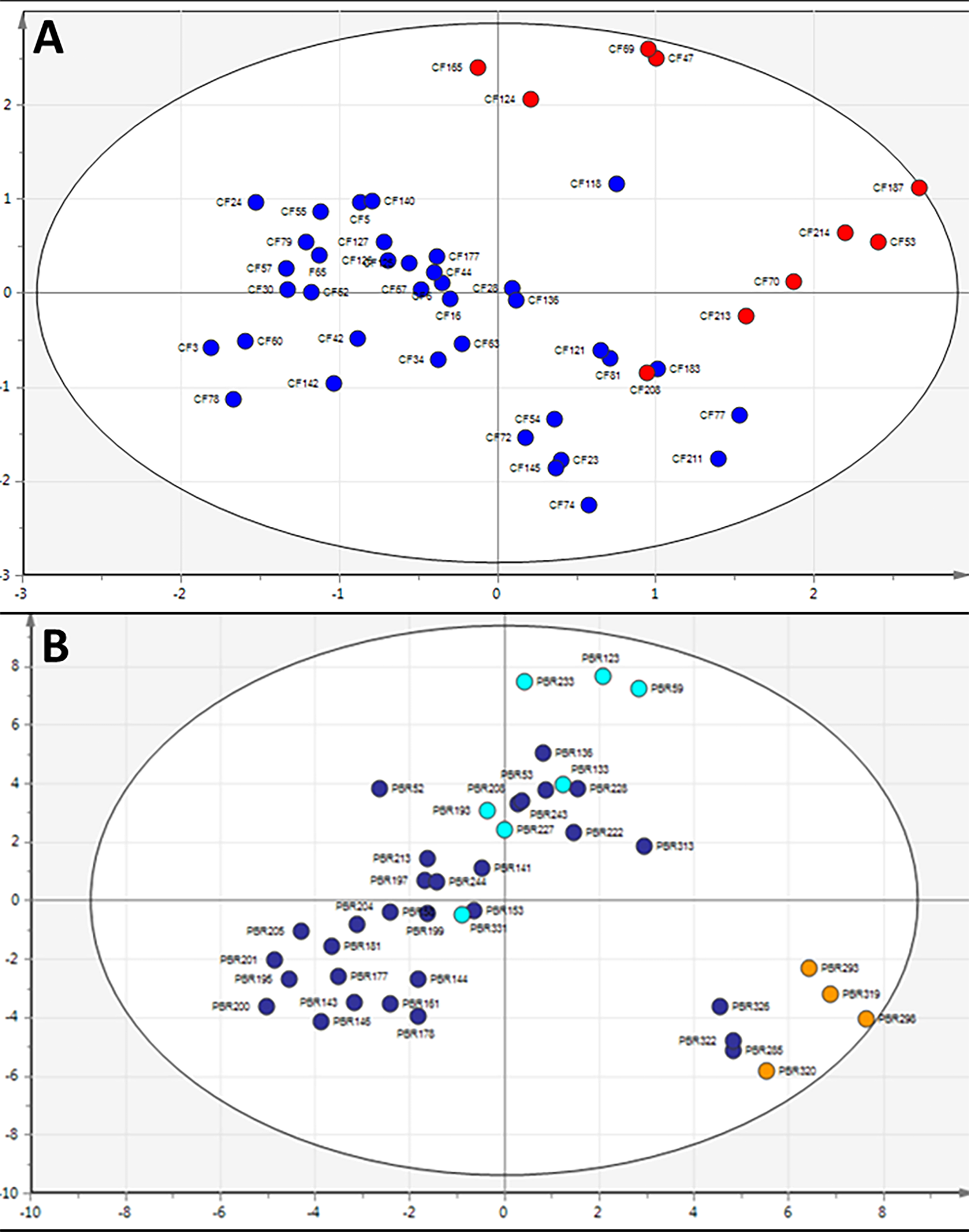

Supplement: FIG S1 [file mSystems.00191-18-sf001.tif]

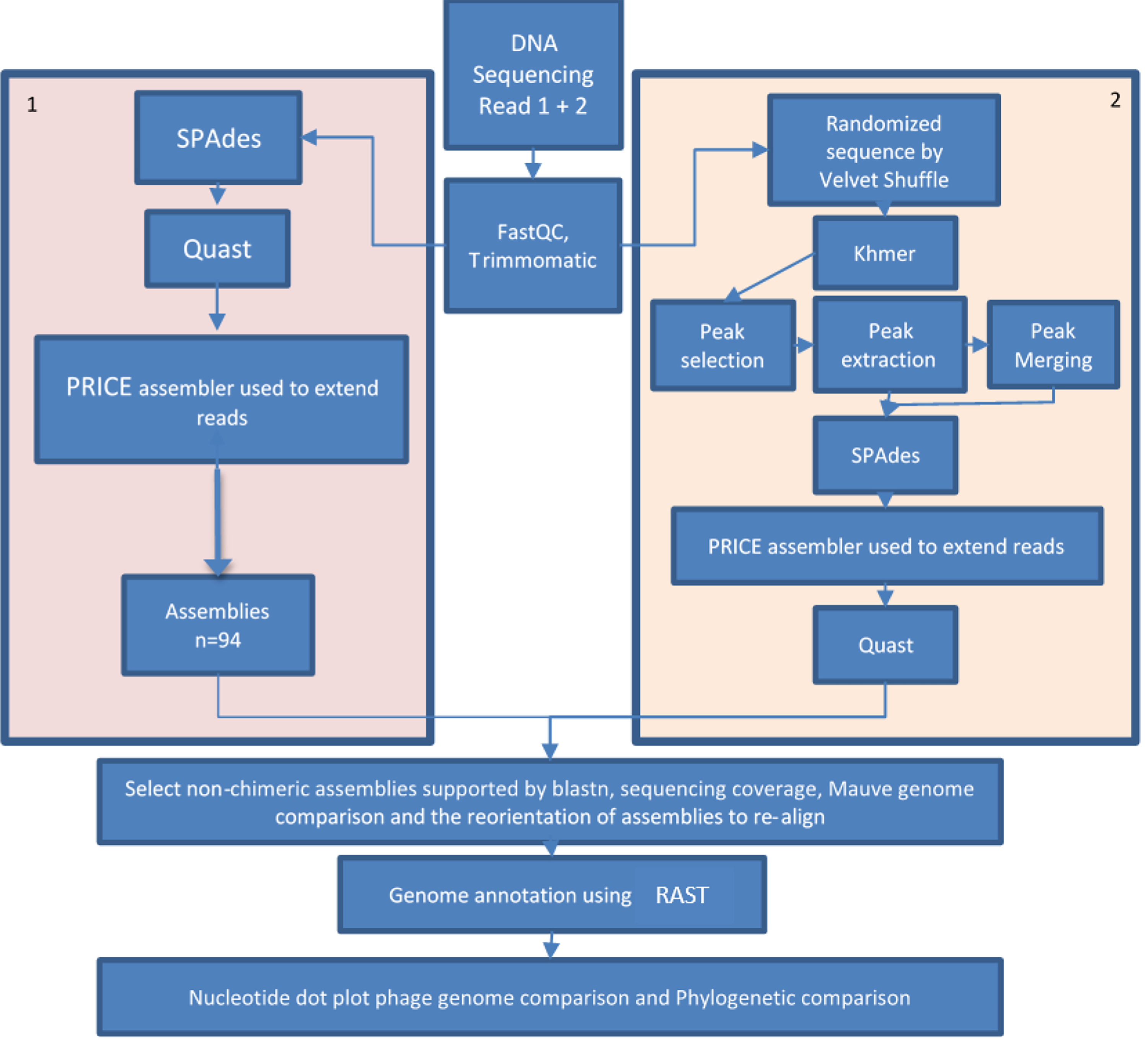

Supplement: FIG S2 [file mSystems.00191-18-sf002.tif]

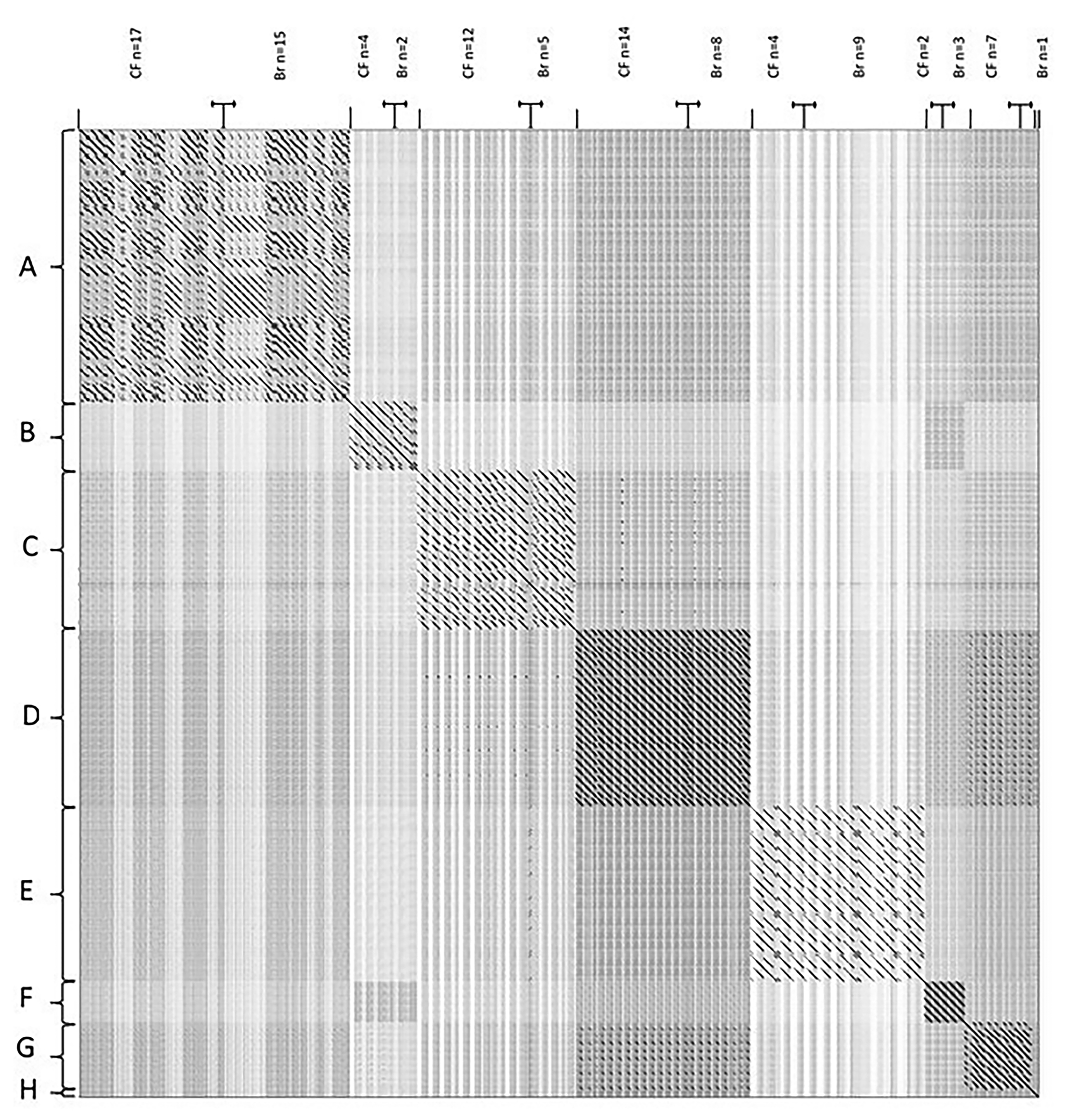

Supplement: FIG S3 [file mSystems.00191-18-sf003.tif]

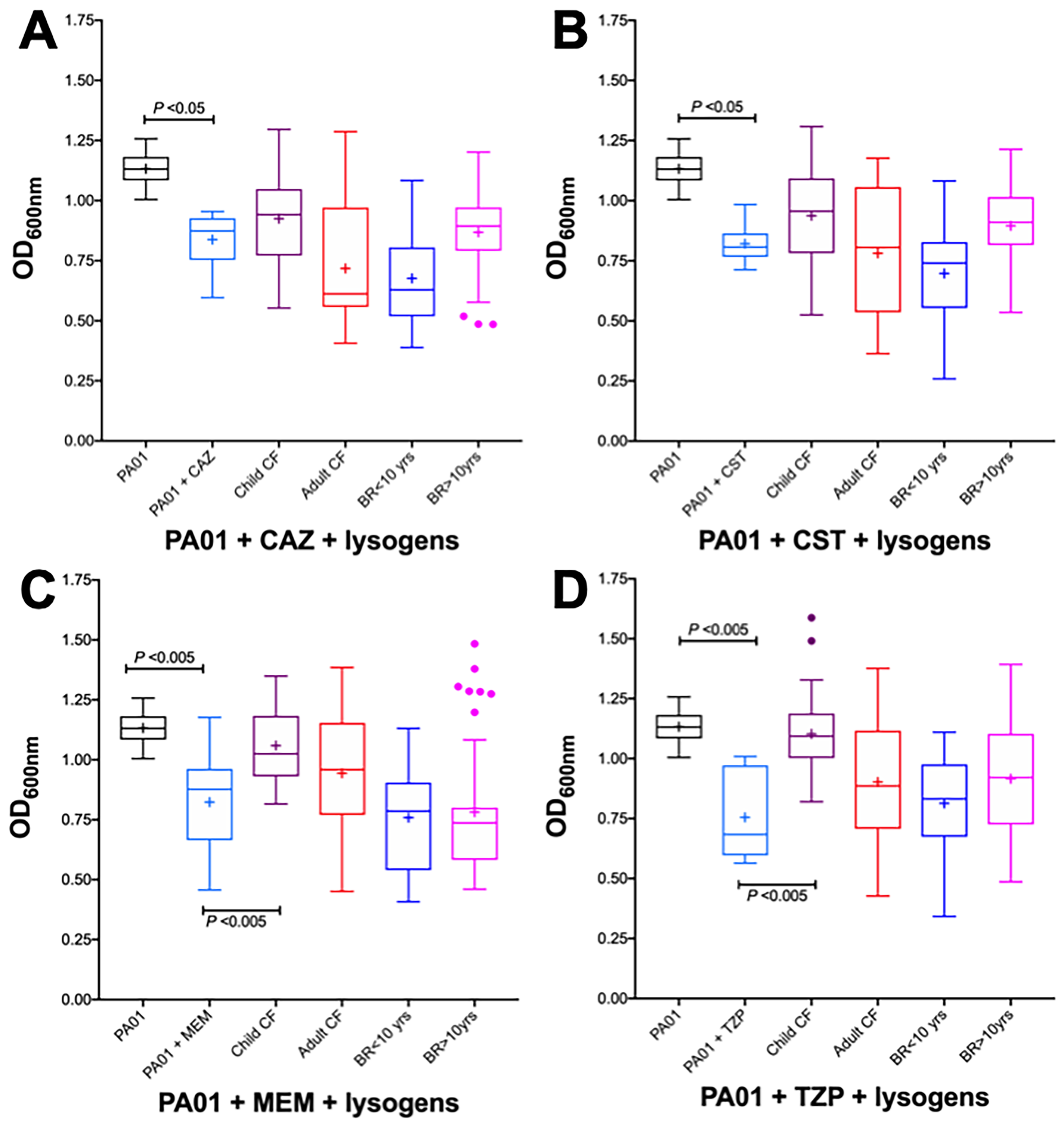

Supplement: FIG S4 [file mSystems.00191-18-sf004.tif]
