# Supplementary material for: Temperate Bacteriophages from Chronic Pseudomonas aeruginosa Lung Infections Show Disease-Specific Changes in Host Range and Modulate Antimicrobial Susceptibility
Source: mSystems. 2019 Jun 4;4(4):e00191-18. doi: 10.1128/mSystems.00191-18 (PMC6550368; doi:10.1128/mSystems.00191-18)
Supplement: TEXT S1 [file mSystems.00191-18-s0001.docx]

**Supplemental material:**

**Mobilizing Temperate Bacteriophage Communities and Resolving Individual Phage Genomes from Cystic Fibrosis and Bronchiectasis *Pseudomonas aeruginosa* Isolates**

Running title: Resolving phage genomes in chronic lung infections

Mohammad A. Tariq^a^, Francesca L. C. Everest^a^, Lauren A. Cowley^b^_,_ Rosanna Wright^c^,

Giles S. Holt^a^, Hazel Ingram^a^, Liberty A. M. Duignan^a^, Andrew Nelson^a^, Clare V. Lanyon^a^ , Audrey Perry^d^, John D. Perry^d^, Stephen Bourke^e^, Michael A. Brockhurst^c^ , Simon H. Bridge^a,f^, Anthony De Soyza^d,f,1^ and Darren L. Smith^a,f,1,2^

^a^ Faculty of Health and Life Sciences, University of Northumbria, Newcastle Upon Tyne NE1 8ST, UK; ^b^ Dept of Biology and Biochemistry, University of Bath; ^c^ Department of Animal and Plant Sciences, University of Sheffield, Sheffield S10 2TN, UK; ^d^ Freeman Hospital, Newcastle Upon Tyne NE7 7DN, UK; ^e^ Royal Victoria Infirmary Hospital, Newcastle Upon Tyne NE1 4LP, UK; ^f^ Institute of Cellular Medicine, Newcastle University, Newcastle Upon Tyne NE2 4HH, UK.

^1^Contributed equally to this work

^2^To whom correspondence should be addressed. Email: darren.smith@northumbria.ac.uk

**SUPPLEMENTARY METHODS:**

**Patient disease etiology and stratification:**

Paediatric CF (Paed CF, age < 16 years, n = 10),

Adult CF (Ad CF, age > 16 years, n = 37),

BR with < 10 years since clinical diagnosis (< 10 years, n = 17; abbreviated to < 10 years BR) and

BR with > 10 years since clinical diagnosis (> 10 years, n = 30; abbreviated to > 10 years BR).

**Partial least squares Discriminant Analysis (PLS-DA) modelling of the cross infection data.**

Data analyses were performed using the SIMCA-P + v 13.0 software (Umetrics, Umea, Sweden). Prior to analysis, the data was centre scaled and treated qualitatively to remove the effects of weighting against a zero value; here indicated no response. The data was analysed using group classification to evaluate the relationships between phage infectivity and bacterial host sensitivity, in relation to patient age, disease etiology, time since diagnosis and bacterial phenotype. The ability to classify each individual in the correct group was assessed by multiple correlation coefficients *R*^2^*Y* and the prediction power of the model was assessed by the *Q*^2^ parameter. Data confidence was assessed using Hotelling’s *T2* tolerance limits (set at 0.95). Outliers that were deemed to be moderate, and unable to shift in the model plane were also subjected to a DModX (Distance to model in the X space) calculation to assess those outliers that did not fit the model well, and deviate from the normal F-distribution (critical distance 0.05).

**Khmer toolkit for k-mer based separation method:**

This method was used to remove very low-level bacterial contamination from the viral sequence data. The k-mer histogram clusters low abundance k-mers that would be linked to any residual bacterial chromosomal DNA. A python v2 script was then used to select abundant viral k-mer sequencing data (20). The following commands were used in order to achieve this in khmer v1.1.

$ load-into-counting.py –k 20 -N 4 -X 4e9 out.kh shufflesequence.fastq

$ abundance-dist.py out.kh shufflesequence.fastq out.hist

For each individual sequence file assessed, the out.hist files were graphed in excel to remove the error k-mer peak. The calc-median-distribution.py script was altered, accordingly to select out the differential phage k-mers associated to the peaks. The script can be found on Github and is renamed k-mer_extraction.py (58).

The script was used, depending on the number of peaks and the k-mer count range were evaluated from the graphed out.hist file.

Using the following command we would generate separate files containing the peak sequences and the peak.hist file was used to create the final k-mer graphs. The output fastq files were then input into the SPAdes assembly program to resolve phage contigs.

$ python k-mer_extraction.py out.kh shuffleSequence.fastq peak.hist
