# Supplementary material for: Temperate Bacteriophages from Chronic Pseudomonas aeruginosa Lung Infections Show Disease-Specific Changes in Host Range and Modulate Antimicrobial Susceptibility
Source: mSystems. 2019 Jun 4;4(4):e00191-18. doi: 10.1128/mSystems.00191-18 (PMC6550368; doi:10.1128/mSystems.00191-18)
Supplement: TABLE S1 [file mSystems.00191-18-st001.docx]

| **Isolate number** | **Percentage infectivity across panel** | **peak1** | **peak2** | **peak3** | **peak4** | **peak5** | **peak6** | Resolved Phage and accession number |
| --- | --- | --- | --- | --- | --- | --- | --- | --- |
| **CF3** | 100 | F10* | F10* |  |  |  |  | vB_Pae_CF3a (MK510962) |
| **CF5** | 100 | B3*, F10 and vB_PaeS_PMG105 |  |  |  |  |  | vB_Pae_CF5a (MK511057) |
| **CF6** | 98 | vB_PaeS_PMG1 | vB_PaeS_PMG105* | F10 |  |  |  | vB_Pae_CF6a (MK510994) |
| **CF16** | 97 | vB_PaeS_PMG105* |  |  |  |  |  | vB_Pae_CF16a (MK511000) |
| **CF23** | 98 | Phi297* and D3112* |  |  |  |  |  | vB_Pae_CF23a (MK511001) and vB_Pae_CF23b (MK511017) |
| **CF24** | 94 | H66* and F10* | D3 |  |  |  |  | vB_Pae_CF24a(MK511039) and vB_Pae_CF24b (MK510963) |
| **CF28** | 99 | D3112* | F10* | Phi297 |  |  |  | vB_Pae_CF28a (MK511018) and vB_Pae_CF28b (MK510964) |
| **CF30** | 99 | F10 | F10 | F10 | F10 |  |  |  |
| **CF34** | 88 | F10* | F10* | F10* |  |  |  | vB_PaeCF34a (MK510965) |
| **CF42** | 97 | MP22 | Novel Phage |  |  |  |  |  |
| **CF44** | 65 | Novel Phage |  |  |  |  |  |  |
| **CF47** | 82 | MP22 |  |  |  |  |  |  |
| **CF52** | 63 | F10 | F10 | PAO1Ab30* and vB_PaeS_PGM105* |  |  |  | vB_Pae_CF52a (MK510995) and vB_Pae_CF52b (MK511052) |
| **CF53** | 80 | F10* and B3* | D3 and Phi297 | D3112* |  |  |  | vB_Pae_CF53a (MK510966), vB_Pae_CF53b (MK511058) and vB_Pae_CF53c (MK511019) |
| **CF54** | 74 | D3 and F10* | D3 and F10* | D3112, Phi297 and JBD24 | D3 |  |  | vB_Pae_CF54a (MK510967) |
| **CF55** | 74 | F10 | F10 | F10* and vB_PaeS_PMG105* |  |  |  | vB_Pae_CF55a (MK510996) and vB_Pae_CF55b (MK510968) |
| **CF57** | 89 | D3112* | Phi297* |  |  |  |  | vB_Pae_CF57a (MK511002) and vB_Pae_CF57b (MK511020) |
| **CF60** | 76 | F10 | F10 | F10 |  |  |  | vB_Pae_CF60a (MK510969) |
| **CF63** | 81 | H66* and PhiCTX |  |  |  |  |  | vB_Pae_CF63a (MK511040) |
| **CF65** | 51 | D3112* | Phi297* |  |  |  |  | vB_Pae_CF65a (MK511003) and vB_Pae_CF65b (MK511021) |
| **CF67** | 19 | F10* | F10* | F10 |  |  |  | vB_Pae_CF67a (MK510970) |
| **CF69** | 63 | H66* | NA |  |  |  |  | vB_Pae_CF69a (MK511041) |
| **CF70** | 67 | MP29 |  |  |  |  |  |  |
| **CF72** | 73 | Novel Phage |  |  |  |  |  |  |
| **CF74** | 85 | D3112* and Phi297* |  |  |  |  |  | vB_Pae_CF74a (MK511022) and vB_Pae_CF74b (MK511004) |
| **CF77** | 49 | D3112* and vB_PaeS_PMG105* | Phi297 |  |  |  |  | vB_Pae_CF77a (MK510997) and vB_Pae_CF77b (MK511023) |
| **CF78** | 31 | B3 |  |  |  |  |  | vB_Pae_CF78a (MK511059) |
| **CF79** | 32 | F10* | F10 | F10* |  |  |  | vB_Pae_CF79a (MK510971) |
| **CF81** | 68 | F10 | D3112* | Phi297* |  |  |  | vB_Pae_CF81a (MK511005) and vB_Pae_CF81b (MK511024) |
| **CF118** | 47 | D3112* | Phi297* | D3112* and Phi297* |  |  |  | vB_Pae_CF118a (MK511006) and vB_Pae_CF118b (MK511025) |
| **CF121** | 72 | Novel Phage and F10* | PAJU2 | D3 | Phi297* | D3112* |  | vB_Pae_CF121a (MK511026), vB_Pae_CF121b (MK511007) and vB_Pae_CF121c (MK510972) |
| **CF124** | 69 | B3 | D3112 | Phi297* |  |  |  | vB_Pae_CF124b (MK511008) |
| **CF125** | 79 | B3 |  |  |  |  |  | vB_PaeCF125a (MK511061) |
| **CF126** | 72 | F116* | PAO1Ab30* |  |  |  |  | vB_Pae_CF126a (MK511042) and vB_Pae_CF126b (MK511053) |
| **CF127** | 54 | F10* | unknown | B3* |  |  |  | vB_Pae_CF127a (MK510973) and vB_Pae_CF127b (MK511062) |
| **CF136** | 55 | D3112* and phi297* |  |  |  |  |  | vB_Pae_CF136a (MK511027) and vB_Pae_CF136b (MK511009) |
| **CF140** | 66 | F10* | F10* |  |  |  |  | vB_Pae_CF140a (MK510974) |
| **CF142** | 62 | JDB24 |  |  |  |  |  |  |
| **CF145** | 37 | F10 | F10* | F10* |  |  |  | vB_Pae_CF145a (MK510975) |
| **CF165** | 44 | F10 and vB_PaeS_PMG105 | F10* | F10* |  |  |  | vB_Pae_CF165a (MK510976) |
| **CF177** | 73 | B3 | D3112* | B3* | Phi297* |  |  | vB_Pae_CF177a (MK511063), vB_Pae_CF177b (MK511028) and vB_Pae_CF177c (MK511010) |
| **CF183** | 27 | Novel Phage | F10* | D3112* | Phi297 |  |  | vB_Pae_CF183a (MK510977) and vB_Pae_CF183b (MK511029) |
| **CF208** | 30 | F10 | F10* | F10* |  |  |  | vB_Pae_CF208a (MK510978) |
| **CF213** | 50 | Phi297* and D3112* | † | † |  |  |  | vB_Pae_CF213a (MK511011) and vB_Pae_CF213b (MK511030) |
| **CF214** | 95 | B3 |  |  |  |  |  |  |
| **BR228** | 46 | H66* |  |  |  |  |  | vB_Pae_BR228a (MK511043) |
| **BR52** | 9 | F10* | D3112* | Phi297 |  |  |  | vB_Pae_BR52a (MK510979) and vB_Pae_BR52b (MK511031) |
| **BR53** | 65 | † |  |  |  |  |  |  |
| **BR59** | 86 | † |  |  |  |  |  |  |
| **BR123** | 67 | F10* | D3112* | Phi297 | vB_PaeSPMG1 |  |  | vB_Pae_BR123a (MK510980) |
| **BR143** | 96 | F10* | Novel Phage, JDB24,D3112 and Phi297 |  |  |  |  | vB_Pae_BR143a (MK510981) |
| **BR152** | 31 | F10 | F10 | F10 |  |  |  |  |
| **BR153** | 53 | Phi297* and JDB25 |  |  |  |  |  | vB_Pae_BR153a (MK511012) |
| **BR161** | 15 | F10* | D3112* | Phi297* | Novel Phage |  |  | vB_Pae_BR161a (MK511013), vB_Pae_BR161b (MK511032) and vB_Pae_BR161c (MK510982) |
| **BR177** | 61 | PAO1Ab30* | F10 | vB_PaeS_PMG105* | F10 | JBD24 |  | vB_Pae_BR177a (MK511054) and vB_Pae_BR177b (MK510998) |
| **BR178** | 16 | F10* | B3 and JBD24 | Phi297 and vB_PaeS_PMG1 |  |  |  | vB_Pae_BR178a (MK510983) |
| **BR193** | 21 | M13 |  |  |  |  |  |  |
| **BR195** | 14 | M13/camphawk |  |  |  |  |  |  |
| **BR197** | 46 | F116* |  |  |  |  |  | vB_Pae_BR197a (MK511044) |
| **BR199** | 21 | M13 |  |  |  |  |  |  |
| **BR200** | 46 | F10 | F10* |  |  |  |  | vB_Pae_BR200a (MK510984) |
| **BR204** | 53 | F10* | PAO1Ab30* |  |  |  |  | vB_Pae_BR204a (MK510985) and vB_Pae_BR204b (MK511055) |
| **BR205** | 21 | D3112* |  |  |  |  |  | vB_Pae_BR205a (MK511033) |
| **BR208** | 6 | H66* |  |  |  |  |  | vB_Pae_BR208a (MK511045) |
| **BR213** | 16 | F10 | F10 | F10* |  |  |  | vB_Pae_BR213a (MK510986) |
| **BR222** | 11 | not enough data |  |  |  |  |  |  |
| **BR227** | 78 | F10 |  |  |  |  |  |  |
| **BR233** | 39 | F10 | F10* | F10* |  |  |  | vB_Pae_BR233a (MK510987) |
| **BR243** | 81 | Novel Phage | Phi297 and D3112 | Phi297* | D3112* |  |  | vB_Pae_BR243a (MK511014) and vB_Pae_BR243b (MK511034) (MK511034) |
| **BR244** | 85 |  | M13 |  |  |  |  |  |
| **BR285** | 82 |  |  |  |  |  |  |  |
| **BR293** | 57 | PAO1Ab30* |  |  |  |  |  | vB_Pae_BR293a (MK511056) |
| **BR298** | 52 | F10 | F10 | F10 |  |  |  |  |
| **BR299** | 90 | F10 | F10 | F10* |  |  |  | vB_Pae_BR299a (MK510988) |
| **BR313** | 54 | F10, D3112*,Phi297* and H66* |  |  |  |  |  | vB_Pae_BR313a (MK511046), vB_Pae_BR313b (MK511035) and vB_Pae_BR313c (MK511015) |
| **BR319** | 52 | PhiCTX* | H66* |  |  |  |  | vB_Pae_BR319a (MK511066) and vB_Pae_BR319b (MK511047) |
| **BR320** | 94 | H66* |  |  |  |  |  | vB_Pae_BR320a (MK511048) |
| **BR322** | 95 | F10 | F10 | F10* |  |  |  | vB_Pae_BR322a (MK510989) |
| **BR326** | 35 | H66* | † | † |  |  |  | vB_Pae_BR326a (MK511049) |
| **BR327** | 78 | F10 | D3112* | Phi297 |  |  |  | vB_Pae_BR327a (MK511036) |
| **BR331** | 29 | † |  |  |  |  |  |  |
| **BR332** | 17 | † |  |  |  |  |  |  |
| **CF187** | 40 | B3 | B3 | B3 |  |  |  |  |
| **CF211** | 68 | JBD24 |  |  |  |  |  |  |
| **BR58** | 63 | B3* | D3112* | D3 | Phi297 | Novel Phage | vB_PaeS_PMG1* | vB_Pae_BR58a (MK511064), vB_Pae_BR58b (MK511038) and vB_Pae_BR58c (MK510999) |
| **BR133** | 82 | F10 | F10 | F10* |  |  |  | vB_Pae_BR133a (MK510990) |
| **BR136** | 84 | DMS3 |  |  |  |  |  |  |
| **BR141** | 50 | F10* | Phi297* | D3112* | Novel Phage B3* | † |  | vB_Pae_BR141a (MK510991), vB_Pae_BR141b (MK511016), vB_Pae_BR141c (MK511065) and vB_Pae_BR141d (MK511037) |
| **BR144** | 57 | F10* |  |  |  |  |  | vB_Pae_BR144a (MK510992) |
| **BR146** | 55 | not enough data |  |  |  |  |  |  |
| **BR150** | 47 | H66* |  |  |  |  |  | vB_Pae_BR150a (MK511050) |
| **BR181** | 13 | H66* |  |  |  |  |  | vB_Pae_BR181a (MK511051) |
| **BR201** | 14 | F10 | F10* |  |  |  |  | vB_Pae_BR201a (MK510993) |
| **BR206** | 19 | F10 |  |  |  |  |  |  |

* Shows the phages that were resolved using >10 sequence coverage and non-chimeric.

† Partial phages and low sequence coverage assemblies.
