# Supplementary material for: Temperate Bacteriophages from Chronic Pseudomonas aeruginosa Lung Infections Show Disease-Specific Changes in Host Range and Modulate Antimicrobial Susceptibility
Source: mSystems. 2019 Jun 4;4(4):e00191-18. doi: 10.1128/mSystems.00191-18 (PMC6550368; doi:10.1128/mSystems.00191-18)
Supplement: TABLE S2 [file mSystems.00191-18-st002.docx]

| **Phage Genotype** | **CF Pediatric (n=10)** | **CF Adult**  **(n=37)** | **< 10 years since diagnosis BR (n=18)** | **> 10 years since diagnosis BR (n=29)** |
| --- | --- | --- | --- | --- |
| **A (F10-like)** | 3 | 14(6) | 5(4) | 10(3) |
| **B (vB_PaeS_PMG105- like)** | 0(1) | 5 | 0(1) | 2 |
| **C (Phi297-like)** | 2(1) | 9(3) | 1(1) | 4(4) |
| **D (D3112-like)** | 2(2) | 12(5) | 3 | 5(3) |
| **E (H66-like)** | 1 | 3 | 3 | 6 |
| **F (PAO1Ab30-like)** | 0 | 2 | 1 | 2 |
| **G (B3-like)** | 1(3) | 6(3) | 1 | 1 |
| **H (PhiCTX-like)** | 0 | 0(1) | 1 | 0 |

Within brackets shows incomplete or partially resolved phage genomes.
