# Supplementary material for: Temperate Bacteriophages from Chronic Pseudomonas aeruginosa Lung Infections Show Disease-Specific Changes in Host Range and Modulate Antimicrobial Susceptibility
Source: mSystems. 2019 Jun 4;4(4):e00191-18. doi: 10.1128/mSystems.00191-18 (PMC6550368; doi:10.1128/mSystems.00191-18)
Supplement: TABLE S3 [file mSystems.00191-18-st003.docx]

| **Lysogen** | **Disease etiology** | **Putative phage** | **Insertion site** |
| --- | --- | --- | --- |
| 47 | Pediatric Cystic Fibrosis | F10-like | Pre-Tyrosine tRNA ligase gene |
| 53 | Pediatric Cystic Fibrosis | F10-like | Pre-Tyrosine tRNA ligase gene |
| 124 | Pediatric Cystic Fibrosis | JBD24-like | Pre-Glycine tRNA gene |
| 165 | Pediatric Cystic Fibrosis | F10-like | Pre-Tyrosine tRNA ligase gene |
| 187 | Pediatric Cystic Fibrosis | H70-like | Pre-HTH-type GntR gene |
| 24 | Adult Cystic Fibrosis | F10-like | Pre-Tyrosine tRNA ligase gene |
| 52 | Adult Cystic Fibrosis | F10-like | Pre-Tyrosine tRNA ligase gene |
| 121 | Adult Cystic Fibrosis | F10-like | Pre-Tyrosine tRNA ligase gene |
| 177 | Adult Cystic Fibrosis | D3112-like | Pre-Tyrosine tRNA ligase gene |
| 74 | Adult Cystic Fibrosis | F10-like | Pre-Tyrosine tRNA ligase gene |
| 152 | < 10 year Bronchiectasis | F10-like | Pre-Tyrosine tRNA ligase gene |
| 233 | < 10 year Bronchiectasis | D3112-like | hpxO1 FAD-dependent gene |
| 299 | < 10 year Bronchiectasis | F10-like | Pre-Tyrosine tRNA ligase gene |
| 327 | < 10 year Bronchiectasis | F10-like | Pre-Tyrosine tRNA ligase gene |
| 332 | < 10 year Bronchiectasis | D3112-like | Pre-Tyrosine tRNA ligase gene |
| 141 | > 10 years Bronchiectasis | D3112 and LPB1 like | Pre-hpxO1 FAD-dependent gene and Pre-Tyrosine tRNA ligase gene |
| 228 | > 10 years Bronchiectasis | LPB1 and D3112-like | Pre-Tyrosine tRNA ligase gene and Pre-HTH-type GntR gene |
| 243 | > 10 years Bronchiectasis | F10-like | Pre-Tyrosine tRNA ligase gene |
| 313 | > 10 years Bronchiectasis | F10-like | Pre-Tyrosine tRNA ligase gene |
| 200 | > 10 years Bronchiectasis | D3112 | Pre-type II secretion pF |

The insertion site is usually between a hypothetical and tyrosine tRNA ligase gene. All phages integrated outside of a putative coding region.
